# Clinical Case Series of Destructive Thyrotoxicosis Associated with Hashimoto’s Thyroiditis Misdiagnosed as Graves’ Disease: Clinical Patterns, Diagnostic Pitfalls, and Hypothesized Molecular Insights

**DOI:** 10.14341/probl13651

**Published:** 2026-07-22

**Authors:** Maher Monir. Akl, Amr Ahmed

**Affiliations:** National Research Lobachevsky State University of Nizhny Novgorod Египет; National Research Lobachevsky State University of Nizhny Novgorod Egypt; The public health department, Riyadh First Health Cluster, Ministry of Health Saudi Arabia

**Keywords:** Thyrotoxicosis Associated with Hashimoto’s Thyroiditis, Graves’ disease, Misdiagnosis, Autoimmune thyroid disorders, Molecular pathways, TRAb testing, Thyrotoxicosis Associated with Hashimoto’s Thyroiditis, Graves’ disease, Misdiagnosis, Autoimmune thyroid disorders, Molecular pathways, TRAb testing

## Abstract

This descriptive clinical case series analyzes five cases of destructive thyrotoxicosis associated with Hashimoto’s thyroiditis, historically referred to as hashitoxicosis, initially misdiagnosed as Graves’ disease, highlighting a persistent diagnostic challenge in autoimmune thyroid disorders. The series includes four published cases reported between 2000 and 2025 and one unpublished case contributed by the authors. The cohort comprised three females and two males, with a mean age of 55.4 years (range: 21–69). Clinical presentations were heterogeneous, most commonly fatigue (80%), palpitations (60%), and weight changes (40%), while two patients exhibited no overt hyperthyroid symptoms.

Biochemical evaluation demonstrated suppressed thyroid-stimulating hormone (TSH) levels in all cases (range: <0.000–0.13 µIU/mL), elevated anti-thyroid peroxidase (anti-TPO) antibodies in 80% (range: 41–>1,000 IU/mL), and initially negative thyroid-stimulating hormone receptor antibodies (TRAb/TSI) in 60% of patients. Seroconversion to positive TRAb/TSI was observed in two cases during follow-up, suggesting autoimmune overlap rather than definitive disease transition. Imaging findings, including thyroid ultrasonography and radioiodine uptake (RAI) studies, consistently favored destructive thyroiditis over stimulatory hyperthyroidism, with heterogeneous echotexture observed in 75% of assessed cases and low or normal RAI uptake in all evaluated patients.

Misdiagnosis occurred in 80% of cases, predominantly due to reliance on suppressed TSH levels without TRAb confirmation, resulting in inappropriate antithyroid drug administration in 80% and accelerated hypothyroidism in 60%. Immunopathological interpretation based on existing literature supports a predominantly Th1-mediated destructive process, in contrast to the Th2-driven antibody-mediated stimulation characteristic of Graves’ disease, with rare Th1-to-Th2 immune shifts reported. Clinical outcomes ranged from spontaneous resolution to surgical intervention.

This case series underscores the importance of mandatory TRAb testing, adherence to American and European Thyroid Association guidelines, and early specialist referral to reduce iatrogenic harm and improve diagnostic precision in autoimmune thyroid disease.

## 1. Introduction

The transient hyperthyroid phase of Hashimoto’s thyroiditis, commonly described as destructive thyrotoxicosis and historically referred to as hashitoxicosis, occurs in approximately 4–10% of affected patients and represents a well-recognized but frequently misinterpreted clinical phenomenon. Hashimoto’s thyroiditis itself is a prevalent autoimmune endocrine disorder affecting an estimated 5–20% of the global population. The hyperthyroid phase arises from immune-mediated follicular disruption, leading to the passive release of preformed thyroid hormones into the circulation rather than increased hormone synthesis [1].

In contrast, Graves’ disease is characterized by sustained thyrotoxicosis driven by thyroid-stimulating hormone receptor antibodies (TRAb), which activate adenylate cyclase and cyclic adenosine monophosphate (cAMP) signaling pathways, resulting in increased de novo thyroid hormone synthesis and follicular hyperplasia [2]. Accurate differentiation between destructive thyrotoxicosis and Graves’ disease is clinically critical, as misdiagnosis has been reported in up to 20–30% of cases in primary care settings, particularly among older adults with subclinical or atypical presentations. Such diagnostic errors frequently result in unnecessary exposure to antithyroid drugs and subsequent iatrogenic hypothyroidism, with potentially serious consequences for patients aged 60 years and older, including increased risks of osteoporosis and atrial fibrillation [3][4]. Measurement of TRAb levels constitutes a cornerstone in distinguishing destructive thyrotoxicosis from stimulatory hyperthyroidism. Negative or low TRAb titers favor a diagnosis of destructive thyrotoxicosis associated with Hashimoto’s thyroiditis, whereas positive titers are strongly indicative of Graves’ disease [5]. Radioiodine uptake (RAI) scintigraphy further aids differential diagnosis, typically demonstrating low or normal uptake in destructive thyroiditis due to follicular damage, in contrast to the diffusely increased uptake observed in Graves’ disease [6].

From an immunopathogenetic perspective, Hashimoto’s thyroiditis is predominantly associated with a Th1-skewed immune response, involving CD8⁺ cytotoxic T lymphocytes and activated macrophages that promote thyrocyte apoptosis through Fas–Fas ligand interactions and caspase activation [7]. This process is amplified by proinflammatory cytokines such as interferon-gamma (IFN-γ) and tumor necrosis factor-alpha (TNF-α), which enhance major histocompatibility complex (MHC) class I and II expression and impair sodium–iodide symporter-mediated iodine uptake [8]. Oxidative stress mediated by reactive oxygen species (ROS) may further exacerbate mitochondrial dysfunction and endoplasmic reticulum (ER) stress, activating the PERK–eIF2α–ATF4 signaling pathway and culminating in C/EBP homologous protein (CHOP)-mediated apoptosis, thereby increasing autoantigen exposure [9][10].

Conversely, Graves’ disease is characterized by a Th2-predominant immune profile, in which interleukin-4 (IL-4) and interleukin-10 (IL-10) promote B-lymphocyte differentiation and TRAb production, leading to sustained hormone synthesis [11]. These fundamentally distinct mechanisms destructive hormone leakage versus antibody-mediated stimulation underscore the importance of precise diagnostic discrimination, as antithyroid drug therapy may exacerbate follicular damage in destructive thyrotoxicosis and accelerate progression to hypothyroidism [12].

Rare instances of antibody profile changes, including reported transitions between Hashimoto’s thyroiditis and Graves’ disease, have been described in the literature and may be temporally associated with immune-modulating events such as vaccination or surgery; however, such observations remain uncommon and should be interpreted cautiously [13]. Despite clear recommendations from the American Thyroid Association (ATA, 2016) and the European Thyroid Association (ETA, 2018), which advocate conservative management with β-adrenergic blockade in transient hyperthyroidism in the absence of high-risk features, diagnostic pitfalls persist in routine clinical practice.

This descriptive clinical case series consolidates four published cases of destructive thyrotoxicosis associated with Hashimoto’s thyroiditis (historically termed hashitoxicosis) misdiagnosed as Graves’ disease between 2000 and 2025, supplemented by one unpublished case. The aim is to delineate recurring clinical patterns, diagnostic challenges, and therapeutic consequences while integrating hypothesized molecular and immunological frameworks derived from existing literature to enhance diagnostic accuracy and promote evidence-based management of autoimmune thyroid disorders.

## 2. Methodology

This study was designed as a descriptive clinical case series combined with a focused narrative review, aimed at systematically analyzing reported instances of destructive thyrotoxicosis associated with Hashimoto’s thyroiditis misdiagnosed as Graves’ disease, with particular emphasis on clinical heterogeneity, diagnostic pitfalls, and therapeutic consequences. A comprehensive literature search was conducted using PubMed, Scopus, Google Scholar, and the Russian Science Citation Index to identify relevant case reports published between January 2000 and August 2025. Search terms included “destructive thyrotoxicosis,” “hyperthyroid phase of Hashimoto’s thyroiditis,” “hashitoxicosis misdiagnosed as Graves’ disease,” “autoimmune thyroiditis misdiagnosis,” and their Russian-language equivalents, combined using Boolean operators (AND, OR).

Five cases were included in the final analysis: four identified from the published literature and one previously unpublished case contributed by the authors. Inclusion criteria comprised documented cases of destructive thyrotoxicosis initially diagnosed as Graves’ disease, supported by serological evidence (TSH, free T3/T4, anti-TPO antibodies, and TRAb/TSI) and/or imaging findings (ultrasonography, scintigraphy, or computed tomography). Exclusion criteria included review articles, cases lacking primary diagnostic data, or reports without clear documentation of initial misdiagnosis.

Data extraction was independently performed by two reviewers using a standardized collection form capturing patient demographics, clinical presentation, laboratory and imaging findings, diagnostic course, therapeutic interventions, and outcomes. Discrepancies were resolved by consensus to ensure data reliability. Quality appraisal of the included reports was conducted in accordance with the CARE (CAse REport) guidelines.

Given the limited sample size (n=5), a narrative synthesis approach was employed to identify recurring clinical patterns, diagnostic errors, and management-related consequences. Hypothesized molecular and immunological interpretations were incorporated exclusively from existing experimental and clinical literature and were not derived from direct molecular analyses of the reported patients. Ethical approval was not required for the published cases, while inclusion of the unpublished case was conducted with documented informed consent and approval from the relevant institutional ethics committee.

The five selected cases were drawn from an initial pool of eleven identified reports and were prioritized based on their capacity to illustrate diverse clinical, immunological, and diagnostic scenarios relevant to the study objectives, including clinical heterogeneity, autoimmune complexity with TRAb/TSI seroconversion, anatomical and diagnostic uniqueness, therapeutic implications, and guideline relevance.

## 3. Cases Presentation

This clinical series encompasses five cases of destructive thyrotoxicosis associated with Hashimoto’s thyroiditis (historically termed hashitoxicosis) initially misdiagnosed as Graves’ disease, comprising four published case reports identified from the literature (2000–2025) and one unpublished case contributed by the authors. Detailed case descriptions, laboratory findings, imaging results, diagnostic errors, management strategies, and outcomes are presented in accordance with CARE guidelines.

All units have been standardized for consistency (e.g., free T4 converted to ng/dL using the conversion factor of 1 ng/dL ≈12.87 pg/mL where applicable). NR = not reported. Reference ranges (standardized across cases for comparability): TSH: 0.4–4.0 µIU/mL; free T3: 2.3–4.2 pg/mL; free T4: 0.8–1.8 ng/dL; anti-TPO: <35 IU/mL; TRAb/TSI: <1.75 IU/L or <140%; anti-Tg: <20 IU/mL; thyroglobulin: 1.6–59.9 ng/mL; RAI uptake: 5–30% (24-hour). Assay methods are specified where reported; standard immunoassays include enzyme-linked immunosorbent assay (ELISA) or chemiluminescence immunoassay (CLIA/ECLIA) variants. The key clinical, laboratory, imaging, treatment, and outcome data of the included cases are summarized in Table 1.

**Table:** Table 1. Summarizes the laboratory parameters, imaging findings, misdiagnoses, treatment approaches, and outcomes of the five identified cases of hashitoxicosis initially misdiagnosed as Graves’ disease. The table highlights the variability in serological markers (TSH, free T3, free T4, anti-TPO, TRAb/TSI), imaging features, and therapeutic responses, illustrating the diagnostic challenges and the clinical course toward hypothyroidism or fluctuating thyroid states in most patients.

Case	Age/Sex	TSH (µIU/mL)	Free T3 (pg/mL)	Free T4 (ng/dL)	Anti-TPO (IU/mL)	TRAb/TSI/TBII	Anti-Tg (IU/mL)	Thyroglobulin (ng/mL)	Method	Imaging Findings	Misdiagnosis	Treatment	Outcome
1 (Current)	60/F	0.13	3.8	1.8	230	Negative	NR	NR	CLIA/ECLIA	US: heterogeneous echotexture with hypoechoic areas, consistent with autoimmune thyroiditis; no diffuse enlargement or increased vascularity	Graves’ disease	Carbimazole 30 mg/day (discontinued upon endocrinology referral); conservative management with β-blockers as needed; serial TFT monitoring every 4–8 weeks	Rapid suppression of thyroid function leading to accelerated hypothyroidism; spontaneous resolution to euthyroid state within 6 months; no long-term complications reported on 1-year follow-up; patient remains stable without levothyroxine replacement
2 (2018)	21/M	0.02	NR	2.0	>1,000	TSI: 164.9	517	NR	Standard immunoassays	RAI uptake: 9.6% (suggestive of destructive thyroiditis); no US reported	Graves’ disease	Methimazole (dose NR) + atenolol; tapered over 16 months; levothyroxine initiated later for hypothyroidism	Persistent hyperthyroidism for 2 years transitioning to subclinical then overt hypothyroidism; euthyroid on levothyroxine maintenance; no long-term complications on 3-year follow-up; the prolonged methimazole use, despite indications of destructive thyroiditis (low RAI uptake), stemmed from initial misdiagnosis as Graves’ disease, leading to accelerated hypothyroidism
3 (2024)	60/F	<0.01	6.5	1.6	46 → 41	TSI: 102% (negative) → 294% (positive)	Negative (<1)	NR	CLIA/ECLIA	US: mild enlargement, heterogeneous echotexture, no increased vascularity; RAI uptake: 15%/25% (initial), later 47%/61%	Initially hashitoxicosis, later Graves’ overlap	Methimazole 10 mg/day with titration + atenolol; total thyroidectomy	Fluctuating thyroid states (hyper-, hypo-, and euthyroid); stabilized on levothyroxine post-thyroidectomy with significant symptom improvement; immune triggers (COVID-19 vaccination and GIST resection) suggested as contributors to antibody conversion; no recurrence on 6-month post-surgical follow-up
4 (2016)	67/F	<0.000	8.46	3.22	NR	TRAb: 19.7	NR	294	Standard immunoassays	US: no thyroid remnant; chest CT: 60×40 mm mediastinal mass; scintigraphy: increased intrathoracic RAI uptake	Graves’ disease in ectopic mediastinal thyroid tissue	Levothyroxine 100 µg/day (discontinued) → methimazole 10 mg/day; surgical resection of mediastinal mass	Thyroid function normalized on methimazole; euthyroid post-resection with benign histology; no further therapy needed; no recurrence or complications on 2-year follow-up
5 (2022)	69/M	0.01 → 0.004	3.35 → 4.58	0.82 → 1.21	715 → 78.66	TRAb: 6.2 → 14.7	NR	NR	Standard immunoassays	US: small thyroid, hypervascular, coarse echostructure, consistent with thyroiditis	Graves’ disease	L-thyroxine 50 → 25 µg/day (stopped); carbimazole 10 mg/day; discontinued Feb 2022	Fluctuating thyroid states with temporary normalization; progressed to hypothyroidism (TSH 9.16 µIU/mL, free T4 0.68 ng/dL) by Dec 2021; long-term outcome: euthyroid without replacement therapy on 18-month follow-up, with stable autoimmune markers and no further fluctuations

## 3.1. Case 1 (Current Case)

A 60-year-old woman presented with mild fatigue in the absence of overt hyperthyroid manifestations such as palpitations, weight loss, or tremor.

Her medical history was unremarkable for thyroid disease, autoimmune disorders, atrial fibrillation, or osteoporosis. Physical examination revealed no goiter, ophthalmopathy, or dermopathy. Laboratory evaluation demonstrated subclinical hyperthyroidism, with a suppressed thyroid-stimulating hormone (TSH) level of 0.13 µIU/mL, free triiodothyronine (T3) of 3.8 pg/mL, and free thyroxine (T4) of 1.8 ng/dL. Anti-thyroid peroxidase (anti-TPO) antibodies were elevated (230 IU/mL), while thyroid-stimulating hormone receptor antibodies (TRAb), measured using CLIA/ECLIA assays, were negative. Thyroid ultrasonography revealed a heterogeneous echotexture with hypoechoic areas, consistent with autoimmune thyroiditis. The patient was initially misdiagnosed with Graves’ disease by a non-endocrinologist and treated with carbimazole (30 mg/day), resulting in rapid suppression of thyroid function. Following referral to an endocrinologist, antithyroid therapy was discontinued, and conservative management consisting of observation with β-adrenergic blockade as needed was recommended, alongside serial monitoring at 4–8-week intervals. Thyroid function normalized spontaneously within six months, and the patient remained euthyroid without levothyroxine replacement at one-year follow-up, illustrating the self-limiting course of hashitoxicosis when managed conservatively.

## 3.2. Case 2 (2018)

A 21-year-old man presented with increased appetite, heat intolerance, fatigue, and excessive sweating over several weeks. He had no prior thyroid or autoimmune disease, although a family history of autoimmunity was noted. Physical examination showed mild anxiety, sinus tachycardia (96 beats/min), mild exophthalmos, lid lag, and a fine tremor. Laboratory testing revealed suppressed TSH (0.02 µIU/mL), elevated free T4 (2 ng/dL), markedly elevated anti-TPO antibodies (>1,000 IU/mL), elevated anti-thyroglobulin antibodies (517 IU/mL), and mildly elevated thyroid-stimulating immunoglobulin (TSI; 164.9). A radioiodine uptake (RAI) scan demonstrated low uptake (9.6%), consistent with destructive thyroiditis.

Despite imaging findings suggestive of hashitoxicosis, the patient was initially diagnosed with Graves’ disease and treated with methimazole and atenolol. Prolonged methimazole therapy (over 16 months) was continued, likely due to the initial diagnostic assumption of stimulatory hyperthyroidism. Over time, thyroid function normalized, allowing gradual discontinuation of antithyroid therapy; however, the patient subsequently developed subclinical hypothyroidism (TSH 4.15 µIU/mL), progressing to overt hypothyroidism (TSH 13 µIU/mL, free T4 0.9 ng/dL). Levothyroxine replacement was initiated, maintaining long-term euthyroidism without complications at three-year follow-up. The low RAI uptake, despite mildly elevated TSI, supported a diagnosis of hashitoxicosis rather than Graves’ disease, underscoring the risk of prolonged antithyroid therapy in destructive thyroiditis [14].

## 3.3. Case 3 (2024)

A 60-year-old woman with a history of hypertension, asthma, low-grade gastrointestinal stromal tumor (GIST; T2NxM0), and hyperaldosteronism was referred for endocrine evaluation. She reported weight gain and palpitations but lacked classical hyperthyroid features such as tremor or ophthalmopathy. Physical examination revealed mild thyroid enlargement without goiter or dermopathy. Initial laboratory testing showed suppressed TSH (<0.01 µIU/mL), elevated free T3 (6.5 pg/mL), free T4 (1.6 ng/dL), mildly elevated anti-TPO antibodies (46 IU/mL), negative TSI (102%), and negative thyroglobulin antibodies. Thyroid ultrasound demonstrated heterogeneous echotexture without increased vascularity, and RAI uptake was within the normal range, findings compatible with hashitoxicosis. She was treated symptomatically with methimazole (10 mg/day) and atenolol. One year later, following Pfizer mRNA COVID-19 vaccination and surgical resection of the GIST, she experienced fluctuating thyroid function states. Repeat evaluation revealed positive TSI (294%) and markedly increased RAI uptake, consistent with Graves’ disease. This serological and functional shift represents a rare phenomenon reported in the literature and may reflect immune perturbation rather than a predictable causal relationship.

Total thyroidectomy was performed, followed by levothyroxine replacement, resulting in stable thyroid function without recurrence at six-month follow-up [15].

## 3.4. Case 4 (2016)

A 67-year-old woman presented with palpitations, fatigue, and unintended weight loss. She had undergone total thyroidectomy seven years earlier for nontoxic multinodular goiter and was maintained on levothyroxine without regular follow-up. Physical examination revealed tachycardia but no cervical mass or ophthalmopathy. Laboratory testing demonstrated suppressed TSH, elevated free T3 and free T4, elevated thyroglobulin, and markedly positive TRAb. Cervical ultrasonography showed no thyroid remnant. Whole-body scintigraphy and chest computed tomography identified a mediastinal mass with increased radioiodine uptake, consistent with ectopic thyroid tissue. Levothyroxine withdrawal did not normalize thyroid hormone levels, excluding iatrogenic thyrotoxicosis. Methimazole therapy normalized thyroid function, and surgical resection confirmed benign ectopic thyroid tissue. The patient remained euthyroid without recurrence at two-year follow-up, emphasizing the diagnostic value of advanced imaging in atypical post-thyroidectomy thyrotoxicosis [16].

## 3.5. Case 5 (2022)

A 69-year-old man with slow-onset type 1 diabetes, secondary adrenal insufficiency, and a family history of Graves’ disease was followed for Hashimoto’s thyroiditis-associated hypothyroidism.

After three years of stable levothyroxine therapy, he developed biochemical hyperthyroidism that persisted despite discontinuation of replacement therapy. Subsequent testing revealed positive TRAb and persistent anti-TPO antibodies. He was initially managed as Graves’ disease with carbimazole, resulting in transient biochemical improvement followed by hypothyroidism. Antithyroid therapy was discontinued, and subsequent follow-up demonstrated stable euthyroidism without replacement therapy over 18 months. This case illustrates diagnostic complexity in autoimmune overlap syndromes and reinforces the need for longitudinal monitoring [17].

## 4. Discussion

This descriptive clinical case series of five patients with destructive thyrotoxicosis associated with Hashimoto’s thyroiditis, historically referred to as hashitoxicosis, misdiagnosed as Graves’ disease highlights a well-recognized yet frequently overlooked diagnostic challenge in clinical endocrinology. The presented cases underscore the complexity and heterogeneity of autoimmune thyroid disease, particularly among older adults and individuals with variable clinical or immunological backgrounds.

Rather than proposing causal mechanisms, this discussion aims to contextualize the observed clinical patterns within established pathophysiological concepts reported in the literature and to emphasize evidence-based diagnostic and therapeutic strategies that may reduce iatrogenic harm. The five cases demonstrated a heterogeneous clinical profile, comprising three females and two males, with a mean age of 55.4 years (range: 21–69 years). Fatigue was the most common presenting symptom (4/5, 80%), followed by palpitations (3/5, 60%), while body weight changes were observed in two cases (40%). Notably, classical hyperthyroid manifestations such as tremor or heat intolerance were absent in two patients, illustrating that the hyperthyroid phase of Hashimoto’s thyroiditis may present with subtle or atypical symptoms, thereby increasing the likelihood of diagnostic error.

Laboratory evaluation revealed suppressed TSH levels in all cases, a nonspecific biochemical finding that frequently prompts an initial diagnosis of Graves’ disease in non-specialist settings. Anti-thyroid peroxidase antibodies were elevated in four of five patients, supporting an underlying autoimmune thyroiditis, whereas TRAb/TSI antibodies were negative or initially negative in three cases. Subsequent seroconversion observed in Cases 3 and 5 highlights the dynamic nature of autoimmune thyroid disease and supports previous reports describing overlapping or evolving immunological phenotypes rather than discrete and mutually exclusive disease entities.

Imaging findings further supported a diagnosis of destructive thyroiditis rather than stimulatory hyperthyroidism. Thyroid ultrasonography most commonly demonstrated heterogeneous echotexture without the marked hypervascularity typically observed in Graves’ disease. When available, radioiodine uptake studies showed low or normal uptake, reinforcing the interpretation that thyrotoxicosis resulted from passive hormone release secondary to follicular destruction rather than increased hormone synthesis.

Misdiagnosis as Graves’ disease occurred in four of the five cases, consistent with previously reported misclassification rates in primary care and non-endocrinology settings. This diagnostic error primarily reflects over-reliance on biochemical thyrotoxicosis without confirmatory TRAb testing or functional imaging. These findings emphasize that suppressed TSH levels alone are insufficient to differentiate between autoimmune thyroid disorders and that integrated serological and imaging assessment is essential for accurate diagnosis.

From a pathophysiological perspective, destructive thyrotoxicosis associated with Hashimoto’s thyroiditis represents a transient hyperthyroid phase driven by immune-mediated follicular injury. Experimental and translational studies suggest a predominantly Th1-mediated immune response involving cytotoxic T lymphocytes, activated macrophages, and proinflammatory cytokines such as interferon-gamma (IFN-γ) and tumor necrosis factor-alpha (TNF-α), leading to thyrocyte apoptosis and hormone leakage. Additional mechanisms, including oxidative stress, mitochondrial dysfunction, endoplasmic reticulum stress, and inflammasome activation, have been proposed in the literature; however, these pathways were not directly evaluated in the present patients and should therefore be regarded as hypothetical explanatory frameworks rather than patient-derived conclusions. To facilitate practical differentiation between destructive thyrotoxicosis associated with Hashimoto’s thyroiditis and Graves’ disease, the key diagnostic, immunological, imaging, and therapeutic characteristics are summarized in Table 2.

**Table:** Table 2. Comparative Features of Hashimoto-Associated Destructive Thyrotoxicosis and Graves’ Disease

Feature	Thyrotoxicosis Associated with Hashimoto’s Thyroiditis	Graves’ Disease
TRAb/TSI	Negative or initially negative (60%)	Positive
Anti-TPO	High (80%, 41–>1,000 IU/mL)	Mild or variable
TSH	Suppressed (100%)	Suppressed
Free T3/T4	High-normal or mildly elevated	Markedly elevated
Ultrasound	Heterogeneous, hypoechoic (75% of reported cases)	Diffuse, hypervascular
RAI Uptake	Low or normal (100% of reported cases)	High
Pathogenesis	Th1-mediated destruction	Th2-mediated stimulation
Management	Observation ± β-blockers	Antithyroid drugs, radioiodine, or surgery

Similarly, the observed transition to TRAb positivity in two cases may reflect immune modulation over time, as previously described in longitudinal studies. Although immune challenges such as infections, vaccination, or surgical interventions have been proposed as potential modulators of autoimmune responses, the present observations are anecdotal and do not establish causality. These associations should therefore be interpreted cautiously.

From a therapeutic standpoint, antithyroid drugs were inappropriately initiated in four cases, leading to accelerated hypothyroidism in three patients. This management approach deviates from established American and European Thyroid Association recommendations, which advocate conservative management with observation and symptomatic treatment, including β-adrenergic blockade, for transient destructive thyrotoxicosis. Beta-blockers were underutilized despite clear symptomatic indications in several patients. These findings underscore the clinical consequences of diagnostic misclassification and highlight the importance of guideline adherence.

The comparative summary presented in Table 2 synthesizes key clinical, serological, and imaging features that may assist clinicians in differentiating destructive thyrotoxicosis associated with Hashimoto’s thyroiditis from Graves’ disease in routine practice. This table is intended as a practical aid rather than a diagnostic algorithm.

This case series emphasizes three clinical imperatives: (1) mandatory TRAb testing to accurately confirm or exclude Graves’ disease, (2) strict adherence to American and European Thyroid Association (ATA/ETA) guidelines to prevent iatrogenic harm, and (3) early referral to endocrine specialists for diagnostically complex cases, particularly in older adults or in patients with autoimmune comorbidities. Future research should further investigate antibody profile dynamics, including TRAb/TSI seroconversion as observed in Cases 3 and 5, as well as potential immune-modulating triggers such as vaccination or surgical interventions, to better predict disease trajectories. By synthesizing clinical, serological, imaging, and hypothesized molecular insights from five diverse cases, this study addresses a gap in the literature and provides a practical framework to enhance diagnostic precision and optimize outcomes in autoimmune thyroid disorders.

## 5. Conclusion

This clinical case series demonstrates that destructive thyrotoxicosis associated with Hashimoto’s thyroiditis, historically referred to as hashitoxicosis, remains a frequent source of diagnostic error when misclassified as Graves’ disease. The findings highlight the limitations of relying solely on suppressed TSH levels and emphasize the critical value of integrated serological assessment, particularly TRAb testing, in conjunction with imaging studies. Although molecular and immunological distinctions between destructive and stimulatory autoimmune thyroid disorders are supported by existing literature, their application in clinical practice should remain interpretative rather than deterministic.

Adherence to ATA and ETA guideline recommendations, mandatory TRAb testing, and careful longitudinal monitoring are essential to avoid unnecessary or harmful interventions and to improve patient outcomes. This series contributes to the growing body of evidence supporting precision-based diagnostic approaches in autoimmune thyroid disease and reinforces the need for heightened clinical awareness of transient destructive thyrotoxicosis within the spectrum of autoimmune thyroid disorders.

## Statements and Declarations

Informed Consent: Prior to the inclusion of these cases in the study, comprehensive information regarding the research objectives and procedures was provided to the patients. Written informed consent was subsequently obtained, including consent for clinical follow-up and for the publication of related data, figures, and laboratory investigations.

Funding; the authors received no financial support for the research, authorship, and/or publication of this article.

Competing Interests: The authors declare that they have no known competing financial interests or personal relationships that could have appeared to influence the work reported in this paper.

Ethical Approval: This study was conducted in full accordance with the ethical principles of the Declaration of Helsinki and complies with the CARE guidelines for case reports. Written informed consent was obtained from the patient, including explicit permission to publish relevant clinical data, laboratory results, and all photographic or imaging materials presented in this manuscript.
